# Alcohol interventions, alcohol policy and intimate partner violence: a systematic review

**DOI:** 10.1186/1471-2458-14-881

**Published:** 2014-08-27

**Authors:** Ingrid M Wilson, Kathryn Graham, Angela Taft

**Affiliations:** Judith Lumley Centre, La Trobe University, 215 Franklin Street, Melbourne, VIC 3000 Australia; Social and Epidemiological Research Department, Centre for Addiction and Mental Health, London, Ontario Canada; Department of Psychology, University of Western Ontario, London, Ontario Canada; Dalla Lana School of Public Health, University of Toronto, Toronto, Ontario Canada; National Drug Research Institute, Curtin University, Perth, Western Australia Australia

## Abstract

**Background:**

Intimate partner violence (IPV) is a significant global public health issue. The consistent evidence that alcohol use by one or both partners contributes to the risk and severity of IPV suggests that interventions that reduce alcohol consumption may also reduce IPV. This study sought to review the evidence for effects on IPV of alcohol interventions at the population, community, relationship and individual levels using the World Health Organization ecological framework for violence.

**Methods:**

Eleven databases including Medline, PsycINFO, CINAHL and EMBASE were searched for English-language studies and grey literature published 1 January 1992 – 1 March 2013 investigating whether alcohol interventions/policies were associated with IPV reduction within adult (≥18) intimate relationships. Eleven studies meeting design criteria for attributing effects to the intervention and ten studies showing mediation of alcohol consumption were included in the review. The heterogeneity of study designs precluded quantitative meta analysis; therefore, a critical narrative approach was used.

**Results:**

Population-level pricing and taxation studies found weak or no evidence for alcohol price changes influencing IPV. Studies of community-level policies or interventions (e.g., hours of sale, alcohol outlet density) showed weak evidence of an association with IPV. Couples-based and individual alcohol treatment studies found a relationship between reductions in alcohol consumption and reductions in IPV but their designs precluded attributing changes to treatment. Randomized controlled trials of combined alcohol and violence treatment programs found some positive effects of brief alcohol intervention as an adjunct to batterer treatment for hazardous drinking IPV perpetrators, and of brief interventions with non-dependent younger populations, but effects were often not sustained.

**Conclusions:**

Despite evidence associating problematic alcohol use with IPV, the potential for alcohol interventions to reduce IPV has not been adequately tested, possibly because studies have not focused on those most at risk of alcohol-related IPV. Research using rigorous designs should target young adult populations among whom IPV and drinking is highly prevalent. Combining alcohol and IPV intervention/policy approaches at the population, community, relationship and individual-level may provide the best opportunity for effective intervention.

**Electronic supplementary material:**

The online version of this article (doi:10.1186/1471-2458-14-881) contains supplementary material, which is available to authorized users.

## Background

The World Health Organization (WHO) defines intimate partner violence (IPV) as ‘any behaviour within an intimate relationship that causes physical, psychological or sexual harm’ [1]. WHO recently estimated the global prevalence of physical and/or sexual IPV to be 30% among ever-partnered women [2]. Thus, IPV is a significant global public health and human rights issue that has damaging effects on the health and well-being of women and children, [3] and significant social and economic costs [4].

Alcohol use, especially heavy drinking and drinking large amounts per occasion, is linked to male-to-female partner violence [5]. Across different cultures, violence is more severe when one or both partners (most often the male partner) has been drinking [6]. Meta-analyses suggest that alcohol plays a causal contributing role in aggression generally; [7] however, the extent to which alcohol’s role in IPV is causal, is complex and contested [8]. In addition, across the globe, IPV is a gendered issue, reflecting the unequal power relationships between men and women. Although under experimental conditions alcohol increases aggression in both men and women, the effect is stronger for men [9] and drinking by men has been shown to play a more important role in IPV perpetration than has drinking by women, [10] reflecting the gendered nature of both problem drinking and IPV.

Alcohol is thought to influence aggressive behaviour through detrimental effects on the drinker’s cognitive executive functioning, [11] and problem-solving abilities, [12] narrowing the focus of attention, [13] increasing their willingness to take risks, [14] and increasing concern about personal power among male drinkers [15]. In the context of an intimate couple, when one of the partners has been drinking, he or she will be less able to address conflicts constructively because of (a) the effects of alcohol on cognitive functioning and problem-solving; (b) the drinking partner may have a disproportionate response to a perceived slight, insult or other apparent wrong done by the partner and be less likely to see the partner’s perspective or the situational and environmental factors that may have affected the partner’s behaviour (because of the narrowing of their focus of attention on a specific action of the partner related to their drinking); (c) the drinking partner may engage in highly provocative or aggressive behaviour without thinking about the consequences of his or her actions because of alcohol’s effects on risk-taking; and, (d) for male partners in particular, perceived slights or aggression by the partner may be interpreted as a threat to their masculinity or social identity generally and therefore require an aggressive response to reassert this identity (see [16, 17]). When both partners have been drinking, the role of alcohol may be even greater [18] because of the potential for alcohol to affect the thinking, perceptions and risk-taking of both partners. That is, both partners are more likely to misperceive the other’s behaviour, be less able to resolve the situation without aggression, and be more likely to engage in risky aggression. Social and cultural perceptions of alcohol can also play a role where the acceptance and tolerance of alcohol-related misbehaviour (including aggression), can influence drinkers’ expectations about their behaviour while drinking [19]. This means that, regardless of the effects of alcohol, some people who have been drinking may intentionally engage in aggression or violence toward an intimate partner because they have the expectation that their behaviour will be excused on the basis that they had been drinking at the time.

**Figure 1:**
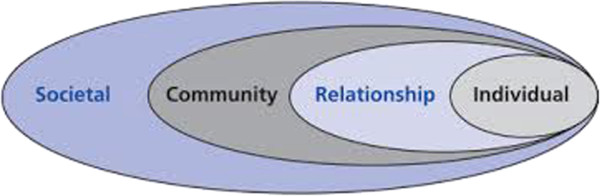
**Ecological model for understanding violence.** Reproduced with permission from the World Health Organization.

Although drinking can occur without IPV and IPV without drinking, both are sufficiently linked that the WHO proposed that primary prevention interventions to reduce the harm caused by alcohol could potentially reduce IPV [20]. Further investigation of the effects of alcohol prevention on IPV is important because direct interventions addressing violence against women have been shown to have limited impact [21]. Recognising the multi-dimensional and complex nature of IPV, the WHO recommends an ecological framework for violence prevention wherein factors influence violent behaviour separately and cumulatively at the individual, relationship, community and societal levels (Figure 1) [1, 22]. Although previous reviews of alcohol interventions have focused exclusively on the individual or relationship level [23, 24] (e.g., individual or couple treatment for alcohol dependency), as this model suggests, alcohol interventions relevant to alcohol-related IPV can occur at the community level and the population level, as well as at the individual/relationship level. Community-level interventions are distinguished from population-level interventions in that they apply to a specific community or area, are often developed in response to local issues or concerns and typically involve community stakeholders in their development and management [25]. Population or societal-level interventions, by contrast, are implemented at the population level more broadly (country, state, region) and may be more likely to involve formal mechanisms such as taxation, although similar interventions can occur at both community and population levels.

Because alcohol use is ‘one of the factors most open to intervention and change,’ [26] (p.viii) and broad evidence exists of effective interventions that reduce alcohol consumption and related harms, [27] this review asks the question: Do interventions to reduce alcohol use at the individual, relationship, community and/or population level, reduce intimate partner violence? In this systematic review, we aimed to explore the evidence for the effects of alcohol interventions on IPV, and the extent to which the effects are mediated by changes in alcohol consumption. For this review, we adopted a broad definition of “intervention” to include interventions specifically implemented to reduce alcohol consumption within a target population or community (e.g., alcohol restrictions or addiction treatment) or alcohol policy levers that may affect alcohol consumption indirectly (e.g., alcohol taxes and planning regulations regarding alcohol outlets).

## Methods

Eleven bibliographic databases were searched systematically for English language peer-reviewed and grey literature studies (such as non peer-reviewed government reports) published between 1 January 1992 and 1 March 2013 including: Medline, CINAHL, EMBASE, PsycINFO, Proquest Central, Cochrane Library, Campbell Collaboration Library, ATSI Health, Drug and Rural Health, and Women’s Studies International. The search strategy combined three concepts of interest: (i) alcohol use, (ii) IPV, and (iii) interventions, using medical subject headings (MeSH), database-specific thesauri search terms, and text-based keywords. Specific terms for alcohol prevention policies were added. Searches were conducted in two stages - the first searches were conducted between 30 October to 1 November 2012 with a second search to update the review conducted on 1 March 2013. A sample search strategy is at (Additional file 1).

A study was included in the review if it investigated whether an intervention or policy to reduce alcohol consumption was directly or indirectly associated with a change in any form of IPV as a primary or secondary outcome. The review included studies of persons 18 years and older and IPV perpetration by either sex within a current heterosexual or homosexual dating, co-habiting or marital relationship, or from a former partner.

**Figure 2:**
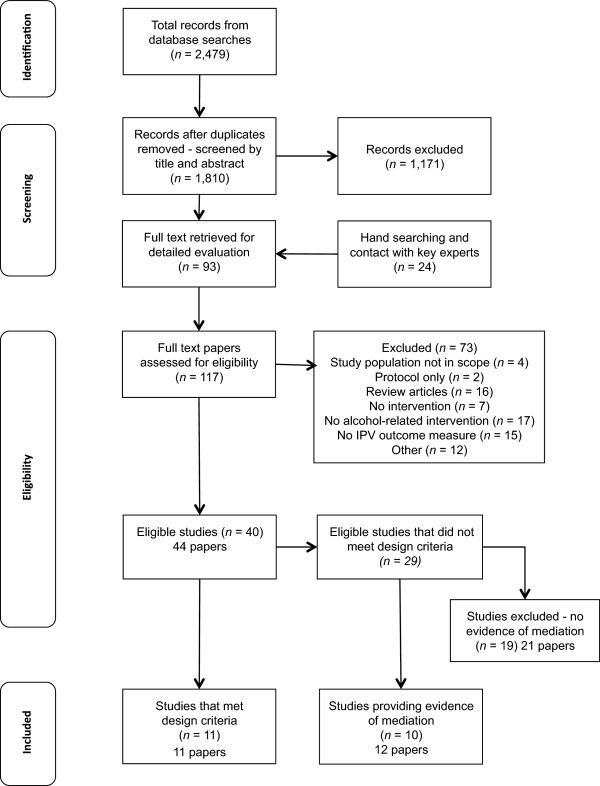
**Selection of articles for review of alcohol and policy interventions to reduce intimate partner violence.**

The search retrieved 1,810 citations as outlined in the flowchart (Figure 2). IW conducted the initial review of study titles and abstracts with 93 (5%) full text papers retrieved. A further 24 papers were identified from hand searching reference lists and contacting key experts. A total of 117 papers were examined against the initial eligibility criteria. Commentaries, reviews or articles that reported no original data were excluded. Due to questions regarding the integrity of research by Dr. William Fals-Stewart (*State of New York v. William Fals-Stewart, 2010*), studies in which he was first author or using his data were excluded.

Forty studies (44 papers) met the initial selection criteria. Data for each study was extracted using an agreed standardised template recording details of the study. This included the PICOS criteria - population/sample, intervention, controls or comparisons, outcomes (IPV and alcohol consumption and other measures pertaining to IPV) and study design. We also noted year of study, aims and strengths and limitations. Two additional criteria were applied prior to final study selection. First, we assessed whether the study design and sample size allowed outcomes to be attributed, at least partly, to the intervention or policy being evaluated. Eleven studies met these design criteria (Additional file 2: Table S1). These included: randomized controlled trials, longitudinal studies that measured IPV over multiple time points before and after the intervention, included multiple replications or used an interrupted time series design. Population and community-level ecological studies that used designs able to rule out explanations other than the intervention to account for changes at the community or population level were included, even when they did not include individual measures necessary for making causal attributions regarding the mechanism of change. Excluded studies used cross-sectional and pre-post designs, small pilot samples and had methodological weaknesses that limited interpretation because of potential bias from regression to the mean and other uncontrolled factors [28].

Because only a small number of studies met the design criteria and many did not test the assumption that the effects of the intervention on IPV were mediated by the intervention’s effect on alcohol consumption, we included in our review a second set of ten studies that support the assumption of mediation (Additional file 3: Table S2). That is, while these studies do not meet the design criterion of being able to rule out other explanations for the apparent effects of the intervention, they do provide some evidence that variations in IPV associated with variations in alcohol consumption and that both are associated with the intervention.

Selected studies were categorised by level in the ecological framework – population, community, relationship and individual-level interventions. IW and AT independently reviewed the individual and couple treatment studies for study strengths and limitations (Additional file 2: Table S1), and IW and KG independently reviewed the population and community-level interventions, with agreement reached by consensus.

In the Results section below, we refer to the 40 studies (44 papers) that met the initial selection criteria but discuss in detail only the findings from the 21 studies that met either the design criteria or provided evidence that the effects of interventions on IPV were mediated through alcohol consumption.

The breadth of the review and the heterogeneity in design and quality precluded formal meta-analysis; therefore, findings were synthesized using a critical narrative approach. This involved considering the theoretical basis for how the intervention might work within each level, critically appraising studies for methodological quality and juxtaposing findings within the ecological framework to draw conclusions across the body of evidence [29].

## Results

### Population-level interventions

#### Alcohol pricing/taxation and IPV

Alcohol consumption is affected by the price of alcohol, which is largely determined by government policy on taxation [27]. Thus, increasing the price of alcohol, either through market forces or taxation, would be expected to reduce the amount of alcohol consumed by those who perpetrate alcohol-related IPV, and by extension the frequency and severity of IPV. Four studies [30–33] evaluated the relationship between alcohol pricing/taxation and IPV. Three met the design criteria [30–32] (Additional file 2: Table S1). The fourth study [33], a pre-post comparison in a single country, was excluded on the basis of design.

Only one study, conducted in the USA, [30] found a significant relationship between the price of alcohol and IPV. Using a composite price of 1 ounce of pure alcohol based on weighted average annual prices of beer, wine and liquor, the study modelled the effects of changes in the price of alcohol on the probability of self-reported ‘husband’ and ‘wife abuse’ from a 1985 national family violence survey and two annual follow ups. The study found that a 1% increase in the price of alcohol was associated with a reduction of 3.1 – 3.5% in wife abuse. No association was found for husband abuse. The study used data from two time points over a three-year period; however, the findings are consistent with cross-sectional comparisons and changes in price were small over that period. The study did not include measures of consumption; thus, it was not possible to assess the mediating effect of consumption.

Of the other two studies of pricing/taxation, one longitudinal study [31] examined the relationship between changes in alcohol taxes, alcohol consumption and female homicide rates across 46 states in the USA (with most women killed by an intimate partner). Their modelling of data from 1990 to 2004 found a significant association between (a) alcohol tax increases and reduced per capita consumption, and (b) reduced consumption and reduced IPV. However, the relationship between alcohol taxes and IPV was not statistically significant, although their results point in this direction. In explaining their results, the authors questioned the extent to which those who consume alcohol *and* commit homicide are sensitive to price.

The third study [32] used a multiple time series design to assess the impact of a range of interventions (including changes to State and Federal beer taxes) on intimate partner homicide (IPH) and IPH involving firearms. Their analysis covered 46 of the largest U.S. cities over 24 years. No relationship was found between increased beer taxes and reduced intimate partner homicide. While the study did not include alcohol consumption measures, the authors suggest that tax increases may have been too small to affect drinking behaviour to the extent needed to influence IPH. Further, their outcome measure included all victims of intimate partner homicide regardless of gender, though evidence shows that women are the overwhelming majority of victims of homicide by an intimate partner [1] and alcohol is more likely to be involved in male-to-female IPV.

In summary, only weak or indirect evidence was found that increasing the price of alcohol reduces IPV.

### Community-level interventions

Alcohol consumption is affected by the physical availability of alcohol as well as other local interventions such as policing and enforcement policies relating to alcohol sales and service [27]. These interventions, such as restricting retail hours or the numbers and density of alcohol outlets within a geographical area, decrease consumption and related harms by increasing the effort to obtain alcohol [34]. Such community-level interventions would be expected to reduce IPV by decreasing drinking opportunities and overall consumption among those who perpetrate alcohol-related IPV.

#### Alcohol sales restrictions and IPV

Only one [35] of eight studies (10 papers) [35–44] that evaluated the impact of community-level restrictions on the hours and days of sale of alcohol on IPV met design criteria for inclusion. The remaining seven studies (9 papers) [28–36] evaluated alcohol restrictions in remote Australian Indigenous communities, with IPV as one of several outcome measures. All were pre-post designs with no comparison group for IPV outcomes. Although some of these studies found decreases in alcohol consumption following the intervention, overall there was no clear pattern of effects on IPV.

The one study with multiple time points [35] examined the effect of a city-wide bar closing time of 11 pm in a mid-sized Brazilian city with high rates of alcohol and violence (Additional file 2: Table S1). Analysing homicide rates over a 10-year period and assaults against women over a 5-year period, this study found that earlier bar closing was associated with a significant reduction in homicides in the first three years post-restriction, and a non-significant reduction in assaults against women. Interpretation is limited by the lesser time period for assaults and the inclusion of all assaults against women, not just IPV. The impact of the intervention on alcohol consumption was not assessed.

#### Alcohol outlet density and IPV

Eleven studies [45–55] conducted in the USA, New Zealand and Australia examined the relationship between alcohol outlet density and IPV. Three studies [45–47] used longitudinal designs (Additional file 2: Table S1). Three cross-sectional studies [48–50] provided additional insight into the possible mediating role of alcohol consumption in the relationship between outlet density and IPV (Additional file 3: Table S2). The remaining five studies [51–55] were cross-sectional designs which revealed inconsistent findings regarding the association between outlet density, type of outlet and IPV.

Among the longitudinal studies, Livingston [45] examined licensing data and police-recorded IPV incidents in Melbourne, Australia, over ten years and found a positive association between IPV and outlet density, with a large and significant effect found for packaged liquor (“off-premises”) outlets. An increase in one packaged liquor outlet per 1,000 residents was associated with a 28.6% increase in the mean domestic violence rate.

A longitudinal study [46] from California using two police-recorded measures of IPV (IPV-related calls to police and crime reports) also found associations with off-premises outlets but no clear association with on-premises outlet density. However, a second study by the same authors [47] using data over a shorter time period found an increased risk of an IPV-related emergency department visit associated with higher on-premises outlet density, while off-premises outlet density was associated with a weaker reduced risk.

In terms of support for the mediating role of alcohol consumption in the relationship between outlet density and IPV, a Western Australian study [48] found a significant association between off-premises sales volume and assaults in private residences, suggesting a potential mediating link between the amount of alcohol sold/consumed (not just number of outlets) and IPV. Similarly, another study using self-reported IPV from a national U.S. survey [49] found a stronger relationship between outlet density and male-to-female physical IPV for couples who had alcohol problems than for couples without. A further study in the U.S. District of Columbia [50] found the association between domestic violence police call-outs and off-premises outlet density was greater for calls on weekends, suggesting links between outlet density and IPV during times when heavier drinking was more likely to occur (i.e., weekends).

Overall, evidence from community studies provides weak support for the association between alcohol availability restrictions and IPV.

### Relationship-level interventions

#### Couples-based treatment

Couple-based alcohol treatment interventions have been shown to be effective for reducing alcohol consumption and improving relationships among treatment-seeking individuals with alcohol and drug problems who are in a married or cohabiting relationship [56]. To the extent that couple-level interventions reduce problem drinking and improve relationship functioning in relationships where there is violence, they may also reduce IPV.

Five studies (seven papers) [57–63] evaluated alcohol interventions involving couples. Of these, only one met design criteria, a trial of a brief intervention [63] (Additional file 2: Table S1) that addressed both IPV and alcohol use. This study assessed an individual motivational feedback session on aggression and IPV risk factors (including alcohol use) among a sample of 49 dating university couples. The study found a greater decrease in harmful alcohol consumption and physical aggression in the intervention group compared with those in the control condition who received minimal non-motivational feedback; however, the reductions in alcohol use and physical aggression were not related.

The remaining four studies did not meet design criteria but did provide some evidence of mediation. These involved pre-post evaluations of behavioral couples-based treatment to address alcohol problems in one partner. These clinical samples were predominantly white, middle-aged and in long term relationships. Three studies reported significant reductions in male-perpetrated violence and verbal aggression between male alcoholics and their female partners, [57–61] and the fourth found decreases in male and female-perpetrated violence in a female alcoholic sample [62]. Although conclusions regarding the effectiveness of the treatment are limited by the single group pre-post design, they did find evidence of a possible mediating role of alcohol consumption. Specifically, increases in violence were found more frequently for relapsed compared with remitted patients, though other explanations for the relationship cannot be ruled out. These studies are shown in (Additional file 3: Table S2).

Overall, other than weak evidence from uncontrolled studies, the only support for the effectiveness of couple interventions focused on alcohol comes from a single well-designed study of a couples-based brief intervention focused on relationship and lifestyle factors, including alcohol use; however, post intervention reductions in alcohol consumption and IPV were not linked. There was evidence of mediation, however, from the uncontrolled studies of persons in treatment for alcohol problems.

### Individual-level interventions

#### Treatment

Individual treatment interventions aim to reduce or eliminate problem drinking in individuals with an alcohol disorder diagnosis or who drink in hazardous or harmful ways. To the extent that their drinking is linked to IPV perpetration, reducing or eliminating alcohol use would be expected to also reduce or eliminate IPV.

Twelve studies [64–75] involved alcohol treatment interventions delivered to individuals. Seven studies of individual alcohol treatment alone on IPV [69–75] did not meet the design criteria. The remaining five studies combined alcohol and batterer treatment using a randomized controlled design [64–68]; however, we excluded two [64, 65] because of small sample size, high attrition rates and lack of power. The three included studies are discussed in more detail below and described in (Additional file 2: Table S1).

Two studies trialled brief interventions. The first, a well-designed randomized controlled trial of a batterer program with a personalised alcohol component, [66] recruited 252 hazardous drinking males enrolled in batterer programs (98% court mandated). The study compared a standard batterer program (SBP) combined with a personalised brief alcohol intervention to an SBP that included one group substance abuse session. The alcohol intervention was a 90 minute therapist-led motivational interview with personalised feedback provided on the participant’s current drinking. The study found significant reductions in the experimental group on drinking outcomes, though these were not sustained. No significant difference was found in the frequency of physical IPV however, the experimental group showed reductions in severe psychological aggression and injuries to partners in secondary analyses. However, improvements dissipated over time.

The second trial [67] assessed a motivational intervention delivered by telephone with substance using IPV perpetrators recruited from the community (i.e., not receiving counselling or legal sanction). The intervention was based on a personalised assessment of IPV and substance use behaviours compared to the control group who received generalised education materials by mail. Less than half the sample (43%) had a diagnosed substance use disorder. At the 30 day follow-up, men in the treatment condition reported engaging in less violence and consumed fewer drinks per week. The authors did not report whether reductions in alcohol consumption were associated with reductions in IPV.

The third study, [68] an integrated substance abuse-domestic violence treatment approach using cognitive behavioral treatment with alcohol dependent men, showed a trend towards greater reduction in IPV and significantly more days abstinent among those in the experimental group compared to a comparison group who received substance-only therapy. However, there were no significant differences at 6 months for either alcohol use or physical IPV.

Of the seven studies of alcohol treatment that did not meet design criteria, three pre-post studies with samples of treatment-seeking male alcoholics [71–73] (Additional file 3: Table S2) found evidence linking alcohol and IPV outcomes, suggesting possible mediation of alcohol consumption in treatment effects on IPV, though design limitations preclude conclusively attributing either outcomes or their relationship to the treatment.

Overall, the evidence for individual-based treatment interventions reducing IPV is limited. Controlled studies of combined alcohol and IPV interventions found significant effects on both behaviors but these effects were not sustained over time and no evidence was provided of mediation. A possible mediating role of alcohol consumption in reductions in IPV was found in three pre-post studies of alcohol treatment alone but the design of these studies precluded the conclusion that these effects were due to the intervention.

## Discussion

For several decades there has been clear and consistent evidence of an association between alcohol consumption and IPV. There is also evidence that alcohol consumption by one or both partners is associated with increased severity of IPV. Thus, interventions that reduce alcohol consumption may also reduce IPV. These interventions can occur at different levels, from individual clinical treatment to state and federal level taxation. An important contribution of the present review is to bring together the disparate literatures relating to alcohol and IPV to examine the effects of alcohol interventions on IPV from the perspective of all levels of the WHO ecological framework - population, community, relationship and individual levels.

Despite the significance of both alcohol misuse and IPV as public health issues, we found the evidence base for assessing the effectiveness of alcohol interventions on IPV from the last 20 years to be disappointingly small. Our review found few studies that had examined the effect of population-level alcohol measures on IPV despite consistent evidence within the alcohol policy science literature that interventions such as alcohol taxation that reduce demand by increasing the cost of alcohol are effective strategies for reducing alcohol consumption and related harms generally [27, 76]. The small literature available suggested little or weak evidence of an effect of alcohol pricing on IPV, possibly hampered by most studies evaluating very small changes in taxation over time and using a measure of IPV that included both alcohol-related IPV and IPV that was not related to alcohol. While all the reviewed studies were based on the theoretical assumption that changes in price influence consumption, only one study [31] included alcohol consumption measures in their design, and it failed to find a strong enough link to demonstrate that alcohol tax changes can reduce violence against women, with this effect mediated through a reduction in alcohol consumption.

To address the effectiveness of alcohol policy more conclusively, stronger designs are needed that evaluate meaningful pricing changes using appropriate comparison conditions, and that include measures that can distinguish effects on *alcohol-related* IPV and measures of alcohol consumption for testing mediation. In addition, given that meta-analyses have shown stronger associations of IPV with heavy and binge-drinking patterns of consumption than with other patterns of drinking [5], it is important to assess the extent that those with heavy or binge drinking patterns are sensitive to changes in alcohol price. To enhance the effectiveness of pricing strategies to reduce alcohol-related harm, such policy approaches should be tailored [77] to suit the patterns of consumption of populations highly likely to engage in IPV, for example, younger couples who are most at risk of IPV [78], and binge drinkers who are most prevalent among adolescents and young adults in many countries [27]. From a public health perspective, population approaches have the opportunity to have the greatest impact; thus, further investigation is warranted regarding how such pricing mechanisms can influence IPV where alcohol use is implicated.

At the community-level, we found policy interventions that restricted the availability of alcohol through reduced trading hours were introduced into communities or areas as a response to significant problems with alcohol and violence. The evidence of an impact on IPV was inconclusive based on the one study in Brazil [35] that met design criteria but which measured violence against women generally, not alcohol-related IPV. The remaining studies of alcohol restrictions were implemented in remote Indigenous Australian communities. Although these studies did not meet design criteria, the comprehensive community approaches used in these studies, with multiple interventions directed toward restricting access to alcohol, provide a model for undertaking better controlled evaluation studies in the future to address alcohol-related IPV, which remains a significant problem in many Indigenous communities worldwide [79].

Although a relatively strong body of research has linked alcohol outlet density to violence, [27] research relating specifically to IPV is inconsistent with regard to outlet type. This finding may reflect a complex and variable relationship between outlet density and IPV. In particular, given that IPV is much more likely to occur in the home than in other locations [80], one might expect a stronger link with drinking in the home and therefore IPV would be more strongly associated with off-premises sales. On the other hand, to the extent that IPV is associated with heavier consumption [78, 81] and heavier consumption is more likely to occur in licensed premises, [82–84] a stronger association might be expected for on-premise drinking. Further, whether the link is with on or off-premise drinking may vary by culture. For example, in some cultures, it may be common for couples to drink together at home with the associated increased risk of aggression, while in other cultures it may be common for the male partner to drink large amounts in licensed premises and become violent after coming home or for conflict to arise over his drinking on his return. Thus, despite this inconsistency in findings related to type of outlet, there is sufficient evidence to suggest that the association between alcohol outlet density and IPV is worth further investigation. Mixed methodology research is needed to better understand the relationship between alcohol-related IPV and drinking location and how this might be linked to outlet density, including the extent to which this association is affected by cultural, social and individual factors not just availability [85, 86].

Over half of the studies we reviewed were treatment studies at the relationship and individual levels of the WHO ecological framework. These studies were all conducted in the USA, the majority of these in clinical settings with older, treatment-seeking alcohol dependent men amongst whom IPV is significantly more prevalent than in the general population [57]. None of the studies of alcohol treatment only - using couple or individual approaches - met design criteria. Thus, although these pre-post studies reported some evidence of reduced IPV after individual or couples-based treatment associated with reduced drinking, excessive drinking and related behaviours such as alcohol-related violence can decrease naturally over time (e.g., natural recovery, regression to the mean) [27]. Well-controlled trials with men in various age groups are needed to confirm that alcohol treatment alone can have an impact on IPV. In addition, while the majority of couples-based alcohol treatment studies measured bi-directional violence, partner substance use was a common exclusion criteria thus the extent to which problematic alcohol use by both partner contributes to IPV, remains untested from the current evidence base.

The more recent treatment studies that combined IPV and alcohol interventions used stronger designs though several had methodological limitations. These studies focused on the effects of the addition of an IPV component to addictions treatment and/or addictions component to IPV treatment to examine the combined effect of addressing both alcohol and IPV. These studies were able to demonstrate significant reductions in alcohol consumption and IPV (compared to the control condition) but these effects were not sustained and none of the studies tested the relationships between reductions in drinking and reductions in IPV. The study of dating couples, [63] though unable to show a link between reductions in alcohol use and physical aggression, suggests that brief interventions with younger, non-dependent adult populations are worthy of further study using larger samples because these are low cost interventions and address the population most at risk in many countries. The randomized controlled trial in which a brief alcohol intervention was added to a batterer program [66] is also important despite changes not being sustained because it is the first of its kind illustrating the potential impact of addressing alcohol within the context of addressing IPV perpetration, an area that has received little attention from the IPV prevention field.

This is the first systematic review to examine studies that have addressed the effects of alcohol interventions on IPV at the population, community, relationship and individual levels. These studies included a variety of research methods from across different disciplines. Despite the importance of this comprehensive approach to examining effects of alcohol interventions on IPV, the existing research on which the review is based has some significant limitations. First, the relatively small literature and the heterogeneity of the study designs precluded meta-analysis or even comparison of effects by characteristics of participants or type of intervention. Second, not all studies included alcohol consumption measures that could be used to test the central assumption that alcohol interventions affect IPV by changing alcohol consumption. Third, most population and community studies used police or hospital statistics to measure IPV which represent the most severe cases of IPV, and none of these studies was able to separate estimates of alcohol-related and non alcohol-related IPV. Thus, one reason for the general null findings of the population and community studies we reviewed could be that a substantial proportion of incidents of IPV do not involve alcohol, with this proportion varying considerably for different countries [6]. Finally, the search strategy focussed on English language studies and interventions found were predominately from middle/high income countries and all studies of couple and individual interventions were from the USA.

## Conclusions

Alcohol-related IPV is a complex, multi-dimensional problem much neglected in intervention and prevention research. Despite the consistent link between alcohol consumption and IPV and evidence that alcohol use contributes to increased risk and severity of IPV, our review found few studies of the effects of alcohol interventions and alcohol policy interventions on IPV where the design allowed changes in IPV to be clearly attributed to the intervention. An appropriately funded research agenda is urgently needed to investigate the potential impact of alcohol/policy interventions on IPV at the population, community, relationship and individual-level, and provide answers to the gaps in the evidence base. This includes:

- better theoretical models of the links between IPV and alcohol consumption, pricing and availability;
- greater focus on those at risk in many countries, such as heavy episodic drinkers and young adults;
- stronger designs, specifically – (i) randomized controlled trials, where possible, or studies with an appropriate comparison group/community, (ii) prospective and longitudinal designs with sufficient statistical power and (iii) designs able to test the mediating role of alcohol consumption;
- more reliable measures distinguishing alcohol-related IPV from IPV not involving alcohol;
- greater consistency of measurement across studies; and
- evaluation of interventions in low and middle income countries where the incidence of IPV is often higher and the link with alcohol stronger [81].

Employing strategies to reduce problematic alcohol use integrated at all levels of the ecological framework (the population, community, relationship and the individual) and combining alcohol and IPV interventions could have the potential to reduce the incidence of IPV and enhance the safety of victims where alcohol use is intertwined with patterns of IPV perpetration.

## Electronic supplementary material

Additional file 1: **Sample Medline search strategy - 1 March 2013.** (DOCX 14 KB)

Additional file 2: Table S1: Studies of alcohol and policy interventions to reduce IPV that met design criteria for assessing the effectiveness of the intervention. (DOCX 31 KB)

Additional file 3: Table S2: Studies of alcohol and policy interventions to reduce IPV that did not meet design criteria but that provided evidence of mediation of alcohol consumption on IPV. (DOCX 26 KB)

## Abbreviations

IPV: Intimate partner violence
WHO: World Health Organization.
