# Supplementary material for: Alcohol interventions, alcohol policy and intimate partner violence: a systematic review
Source: BMC Public Health. 2014 Aug 27;14:881. doi: 10.1186/1471-2458-14-881 (PMC4159554; doi:10.1186/1471-2458-14-881)
Supplement: Supplementary file 1 — Additional file 1: Sample Medline search strategy - 1 March 2013. (DOCX 14 KB) [file 12889_2014_7007_MOESM1_ESM.docx]

**Additional file 1 - Sample Medline search strategy - 1 March 2013**

1. Family Conflict/

2. Courtship/

3. Spouse Abuse/

4. Battered Women/

5. Sexual Partners/

6. ("couples" or "dating couple*" or "dating violence" or "courtship" or "couple violence" or "intimate partner violence" or "domestic violence" or "domestic abuse" or "domestic assault" or "marital abuse" or "spous* abuse" or "domestic abuse" or "domestic violence" or "partner violence" or "family violence" or "domestic assault" or "spous* assault" or "partner violence" or "partner assault" or "partner abuse" or "battered wife" or "battered wives" or "battered women" or "battering" or "wife abuse" or "partner aggression" or "conflict tactics scale" or "composite abuse scale" or "physical abuse").mp. [mp=title, abstract, original title, name of substance word, subject heading word, keyword heading word, protocol supplementary concept, rare disease supplementary concept, unique identifier]

7. 1 or 2 or 3 or 4 or 5 or 6

8. Alcohol Drinking/

9. alcoholic intoxication/ or alcoholism/

10. ("alcoholic use" or "alcohol misuse" or "alcohol consumption" or "alcohol* intoxication" or "alcohol dependence" or "alcohol abuse" or "alcohol addiction" or "alcohol over-consumption" or alcoholic or alcoholism or "binge drink*" or "harmful drink*" or "problem drink*" or "hazardous drink*" or "high risk drink*" or "risky drink*" or "heavy episodic drink*" or "risky single occasion drinking" or intoxication or drunk*).mp. [mp=title, abstract, original title, name of substance word, subject heading word, keyword heading word, protocol supplementary concept, rare disease supplementary concept, unique identifier]

11. 8 or 9 or 10

12. 7 and 11

13. limit 12 to (english language and yr="1992 -Current")

14. primary prevention/ or secondary prevention/ or tertiary prevention/

15. Harm Reduction/

16. Intervention Studies/

17. (prevention or "primary prevention" or intervention or "early intervention" or evaluation or strategy or policy or "harm reduction" or "harm minimisation").mp. [mp=title, abstract, original title, name of substance word, subject heading word, keyword heading word, protocol supplementary concept, rare disease supplementary concept, unique identifier]

18. 14 or 15 or 16 or 17

19. ("alcohol policy" or "liquor policy" or "alcohol pric*" or "liquor pric*" or "alcohol tax*" or "liquor tax*" or "alcohol trading hours" or "liquor trading hours" or "alcohol hours of sale" or "liquor hours of sale" or "trading hours" or "outlet density" or "alcohol outlet density" or "liquor outlet density" or "alcohol marketing" or "liquor marketing" or "alcohol advertising" or "liquor advertising" or "alcohol promotion" or "liquor promotion" or "alcohol availability" or "liquor availability" or "social marketing" or "community action" or "community intervention").mp. [mp=title, abstract, original title, name of substance word, subject heading word, keyword heading word, protocol supplementary concept, rare disease supplementary concept, unique identifier]

20. 18 or 19

21. 13 and 20
