# Supplementary material for: Alcohol interventions, alcohol policy and intimate partner violence: a systematic review
Source: BMC Public Health. 2014 Aug 27;14:881. doi: 10.1186/1471-2458-14-881 (PMC4159554; doi:10.1186/1471-2458-14-881)
Supplement: Supplementary file 2 — Additional file 2: Table S1: Studies of alcohol and policy interventions to reduce IPV that met design criteria for assessing the effectiveness of the intervention. (DOCX 31 KB) [file 12889_2014_7007_MOESM2_ESM.docx]

**Additional file 2: Table S1. Studies of alcohol and policy interventions to reduce IPV that met design criteria for assessing the effectiveness of the intervention**

| **Author (date), country, study design** | **Study aim** | **Description of intervention and measures** | **Population/sample** | **Reported results for IPV, alcohol use, other relevant outcomes and mediation** | **Strengths and limitations** |
| --- | --- | --- | --- | --- | --- |
| **Population-level interventions: Alcohol taxation** | | | | | |
| Markowitz (2000)^30^; USA; repeated measures/cross-sectional design | To examine the direct relationship between the price of alcohol and violence towards husbands and wives across different states. | ***Intervention:*** Changes in the price of liquor, wine and beer as measured by the weighted average of the price of pure alcohol assigned to each person based on the state in which they live.  ***IPV measure:*** Self-reported husband and wife abuse (CTS) from 1985 National Family Violence Survey (NFVS) and 1986 and 1987 follow ups. | National representative population, prices calculated by state. | A 1% increase in the price of pure alcohol was associated with 3.1-3.5% reduced probability of severe wife abuse. No association for violence by wives towards husbands.  ***Other outcomes:*** No relationship between the availability of alcohol (number of outlets) and probability of wife abuse. | Adjusted for socio-economic and demographic factors.  Only included in analysis married or concurrently cohabiting couples.  Did not assess drinking pattern of individual as possible mediator.  Limited changes in NFVS and pricing data suggests effect found is weak. |
| Durrance et al. (2011)^31^; USA; longitudinal design | To evaluate the relationships between alcohol taxes, alcohol consumption and violence towards women. | ***Intervention:*** Changes in State-level beer, wine and liquor taxes between 1990-2004, and increase in Federal-level beer, wine and liquor (spirits) tax from 1991- 2004.  ***IPV measure:*** State-level female homicide victimization rates from 1990-2004.  ***Alcohol consumption:*** per capita consumption from alcohol sales data. | Forty-six U.S. states and the District of Columbia. | No direct link between alcohol taxes and female homicide.  Other outcomes: Increase in beer and wine taxes associated with reduction in per capita beer and wine consumption. Changes in liquor (spirits) tax did not affect liquor consumption.  ***Mediation:*** 1% reduction in per capita consumption associated with 1.33% decline in female homicide rates | Controlled for important confounders. |
| Zeoli and Webster (2010)^32^; USA; multiple time series design | To assess the relationships between intimate partner homicide and relevant public policies (including alcohol taxes) in large U.S. cities between 1979 and 2003. | ***Intervention:*** Changes in Federal, State and local beer excise taxes as measured by alcohol tax index (and other public policies relating to domestic violence – only alcohol-related results described here).  ***IPV measure:*** Intimate partner homicide (IPH) and firearm IPH for period 1979 to 2003 obtained from FBI Supplementary Homicide Reports. | Forty-six of the largest U.S. cities. | Increase in Federal beer taxation and increases in State beer excise not associated with IPH or firearm IPH. | Strong design for detecting impact of policy changes; controlled for important confounders.  Measure of IPH included homicides of both gender though females are majority of homicide victims by intimate partner.  Lack of association may have been affected by low variability in taxation (only 14 of 27 states and 2 cities changed taxation level). |
| **Community-level interventions: Alcohol restrictions** | | | | | |
| Duailibi et al. (2007)^35^; Brazil; longitudinal design | To investigate whether limiting the hours of alcoholic beverage sales in bars had an effect on homicides and violence against women in a Brazilian city. | ***Intervention:*** Introduction of licensing law closing all bars at 11pm in Diadema, São Paulo, Brazil from July 2002.  ***IPV measure:*** Police-recorded assaults against women for period 2000 to 2005.  ***Other:*** Police-recorded homicide data for period 1995 to 2005. | City of Diadema, São Paulo, Brazil, population approximately 360,000. Industrial city, predominantly low socioeconomic status. One of highest homicide rates in Brazil (103 per 100 000 inhabitants) of which 65% alcohol-related. High rate of assaults and most murders of women in or close to bars between 11pm – 6am. | Non-significant reduction (17%) of assaults against women following intervention, 176 assaults (95% CI: -239, 590).  ***Other outcomes:*** Significant decrease (44%) in homicides following intervention, 319 homicides (95% CI: 193, 445). | Could not control for local demographic, social and economic changes due to data limitations.  Proportion of assaults against women perpetrated by intimate partners v non-intimate partners is not specified.  Generalizability only to cities with similar demographics and level of alcohol-related violence. |
| **Community-level interventions: Alcohol outlet density** | | | | | |
| Livingston (2011)^45^; Australia; longitudinal time series design | To assess how changes in postcode-level outlet density related to changes in domestic violence rates over a 10-year period (1996-2005). | ***Intervention:*** Changes in in the number and density of alcohol outlets. Geographical unit: postcode.  ***IPV measure:*** Police-recorded domestic violence incident data for period 1995 to 2005. | Melbourne, Australia (city of Melbourne and suburbs - area covering 5,600m^2^, approximately 3,350,000 residents). | Increase in outlet density associated with a small increase in domestic violence incidents recorded by police; strong association with packaged liquor (off-premises) outlets. An increase of one package outlet per 1,000 residents associated with 28.6% increase in domestic violence rate (*B*=1.36, *p*< 0.01). | Strong design with long time period enabling assessment of changes over time. Controlled for neighborhood socioeconomic and demographic characteristics.  Controlled for spatial autocorrelation.  IPV measure (police-reported IPV) likely to under-represent incidence of IPV. |
| Cunradi et al. (2011)^46^; USA; longitudinal design | To determine if changes in alcohol outlet density are related to changes in rates of IPV-related police calls and IPV-related crime reports in Sacramento, California. | ***Intervention:*** Changes in the number and density of alcohol outlets.  ***IPV measures:*** IPV-related police calls for period 2006 to 2009 (including calls coded for physical violence and verbal altercation). IPV-related crime reports for period 2001 to 2009. | City of Sacramento, California, USA, population approximately 463,794 (2008). | Increase in off-premises alcohol density associated with an increase in IPV-related police call outs. An additional off-premises alcohol outlet associated with 4% increased risk of an IPV call out RR 1.04 (95% CI: 1.01, 1.07) and 3% for IPV-related crime reports, RR 1.03 (95% CI: 1.00, 1.06). No clear association with on-premises outlets. | Controlled for neighbourhood socioeconomic and demographic characteristics.  Controlled for spatial autocorrelation.  Limited to one urban area.  IPV measure likely to underrepresent the incidence of IPV. |
| Cunradi et al. (2012)^47^; USA; longitudinal design | To examine the relationship of outlet density and IPV-related emergency department (ED) visits in California. | ***Intervention:*** Changes in the number and density of alcohol outlets.  ***IPV measure:*** Half yearly counts of IPV-related ED visits for period 2005 to 2008. | State of California, USA. | Increase in on-premises alcohol density (bars and pubs) associated with an increase in IPV-related ED visits. An increase of one on-premises outlet per square mile associated with 3% increased risk of ED visit, RR 1.030 (95% CI: 1.02, 1.05). An increase of one off-premises outlet density had weaker association with decrease in risk of ED visit for IPV, RR 0.99 (95% CI: 0.982, 0.999). | Controlled for neighbourhood socioeconomic and demographic characteristics.  Controlled for spatial autocorrelation.  IPV measure (ED visits) likely to represent more severe IPV resulting in physical injury. |
| **Relationship-level interventions: Couples-based treatment** | | | | | |
| Woodin and O’Leary (2010)^63^; USA; RCT | To examine the effectiveness of motivational interviewing as a targeted prevention approach for partner aggression in emerging adulthood. | ***Intervention:*** Individualized motivational feedback (45 mins) targeting physical aggression and risk factors (including alcohol use).  Control: Minimal, non-motivational feedback (10 mins).  3-, 6- and 9-months follow up.  ***IPV measures:*** Self-reported psychological and moderate physical partner aggression (CTS2).  ***Alcohol use:*** AUDIT. | 49 dating college couples with male perpetration of physical aggression. Recruited from one university site via advertising.  Mean age for women 19.64 (SD=1.26) and men 20.28 (SD=1.42)  Average relationship length 21.47 months (SD=18.37). | ***IPV outcomes:*** Significant overall reduction in physical aggression perpetration over time (effect size *d*=0.58, *p*<0.05) but intervention group reduced their physical aggression at a significantly greater rate than the control group (*d*=0.56, *p*<0.05).  ***Alcohol outcomes:*** Reduction in harmful alcohol consumption in intervention group *(d=*0.70, *p*<0.05).  ***Mediation:*** Reduction of alcohol use was not related to changes in physical aggression. | Non-treatment seeking sample.  Used any aggression reported by either partner to minimize under-reporting.  No description of randomization.  Small sample. No power calculation.  Particularly low follow up participation by male partners. |
| **Individual-level interventions: Treatment** | | | | | |
| Stuart et al. (2013)^66^; USA; RCT | To examine whether adding adjunctive alcohol intervention to batterer intervention reduced both substance use and violence compared to batterer intervention alone. | ***Intervention:*** Standard Batterer Program (40 hours) plus one-off 90-minute motivational alcohol intervention (SBP+AI) (*n*=123)  Control: Standard Batterer Program which included one session on substance use and violence (SBP) (*n*=129)  3-, 6-, and 12-month follow up  ***IPV measures:*** Self-reported frequency of any physical violence (primary outcome) and psychological aggression (CTS2); arrest records for any IPV for the 12 months following intervention.  ***Alcohol use:*** Primary substance use outcome = drinks per drinking day (DPDD) measured by self-report (TLFB); percentage of Days Abstinent from Alcohol (PDAAD); percentage Heavy Drinking Days (PHDD). | 252 hazardous drinking men in batterer intervention programs recruited from 5 sites.  98% court ordered.  Mean age: Intervention group: 31.5 years (SD 9.6); Control group: 31.6 years (SD 9.9).  Relationship length: Intervention group: 5.5 (SD 5.9); Control group: 5.4 (SD 5.3).  Ethnicity: White - Intervention group 71.5%; Control group: 72.1%. | ***IPV outcomes:*** No significant differences between groups in physical IPV. Secondary analyses, intervention group reported less severe physical aggression (Incidence Rate Ratio=0.18, (95% CI: 0.05, 0.65, *p*=0.009) at 3 months, but not 6- or 12-months; less severe psychological aggression at 3 months (B= -1.24, 95% CI: -2.47, -0.02, *p*=0.01); and fewer injuries to partners at 3- and 6-month follow up. (IRR= 0.33, 95% CI: 0.12, 0.92, *p*=0.03).  ***Alcohol outcomes:*** Intervention group reported consuming fewer DPDD at 3-months than control (B= -1.36, 95% CI: -2.65, -0.04, *p*=0.04) but not 6- and 12-months; significantly greater abstinence at 3-months (B=0.09, 95% CI: 0.03, 0.14, *p*=0.002) and 6-months (B=0.06, 95% CI: 0.01, 0.11, *p*=0.01) but not at 12-months.  ***Mediation:*** Changes in alcohol consumption coincided with changes in IPV. | Sample size calculations showed adequate power for alcohol use outcomes but limited power to detect effects for IPV; reduced sample size as a result of 28% of relationships ending during 12-month follow up.  Urn randomization.  Good retention rates that dropped slightly at 12-months.  Intent to treat analysis.  No description of how missing data were accounted for.  Tested adjustment for clustering in five sites.  Did not use partner corroboration of violence and substance use. Acknowledged that arrest not good equivalence for IPV. |
| Mbilinyi et al. (2011)^67^; USA; RCT | To evaluate telephone-delivered motivational enhancement therapy in motivating entry into treatment among non-mandated and nontreatment seeking intimate partner violence perpetrators who also used substances. Impact of intervention on IPV frequency and substance use measured as secondary outcomes. | ***Intervention:*** Personalised motivational enhancement therapy (MET) delivered by telephone (60-90mins feedback session) (*n*=49).  Control: Education materials delivered by mail (*n*=66).  1-week, 30 day follow up.  ***IPV measures:*** (secondary outcome). Self-reported physical/injurious behavior and psychological abuse (CTS2).  ***Alcohol use:*** Self report (Daily Drinking Questionnaire). | 124 male IPV perpetrators recruited from the community through media advertising.  134 eligible, 124 randomized, 9 did not complete MET.  43% had substance use disorder.  Mean age= 39.4 years  Ethnicity: 65% White/ Caucasian; 35% men of color. | ***IPV outcomes:*** Men receiving MET reported engaging in IPV less frequently at 30-day follow-up compared to control group.  ***Alcohol outcomes:*** Follow-up substance use (43% of sample) was strongly associated with baseline substance use and no relationship with intervention condition. Average number of drinks was lower at follow up than at baseline but authors note caution with interpreting these findings because alcohol use was considerably skewed. | Non-mandated, non-treatment seeking population.  Good retention rates with only small loss to follow up (intention-to-treat analysis).  Short follow-up period.  Reliance on self-reported data.  No partner corroboration for IPV. |
| Easton et al. (2007)^68^; USA; RCT pilot | To evaluate the efficacy of a twelve-session cognitive behavioral group therapy for alcohol-dependent males with co-occurring interpersonal violence. | ***Intervention:*** 12-week group-based cognitive behavioral treatment integrating Substance Abuse-Domestic Violence Treatment Approach (SADV) (*n*=40).  Control: 12-week Twelve Step Facilitation (TSF) (*n*=38).  12-week, 6-months follow up.  ***IPV measures:*** Self-reported physical violence (CTS2) and collateral reports from female partners (55%).  ***Alcohol use:*** Self-report (TLFB), breathalyzer, and urine toxicology. | 85 alcohol dependent males arrested for domestic violence.  Recruited from substance abuse outpatient treatment.  78 randomized, 75 started treatment, 62 completed (79% retention).  Mean age = 38 years  Ethnicity: 49% Caucasian, 33% African American, 10% Hispanic. | ***IPV outcomes:*** Trend for greater reductions in frequency of violent episodes for participants in SADV condition compared to TSF group (F=3.3, *p*<0.09). No significant difference between groups at 6 month follow-up.  ***Alcohol outcomes:*** SADV group had significantly more days abstinent compared to controls during treatment period (F= 5.4*, p*<0.02). No significant difference on breathalyzer and urine toxicology and no between group differences at 6 months.  ***Mediation:*** SADV group showed greater improvement in both alcohol consumption and IPV, although both effects had disappeared at 6 months. | Objective measures of substance use.  Corroboration of IPV self-report by female partners (55%).  Urn randomization by computer.  Small sample. No power calculation.  Groups differed at baseline on key variables including physical violence (intervention group reported more physical episodes at baseline F=3.33, *p*<0.06), marital status, prior alcohol treatment and years of marijuana use. Majority of TSF group living alone and no intact relationship. |

AUDIT – Alcohol Use Disorders Identification Test

CTS – Conflict Tactics Scale

CTS2 – Revised Conflict Tactics Scale

TLFB – Timeline Follow Back Interview

TLFB-SV – Timeline Follow Back Interview for Spousal Violence
