# Supplementary material for: Alcohol interventions, alcohol policy and intimate partner violence: a systematic review
Source: BMC Public Health. 2014 Aug 27;14:881. doi: 10.1186/1471-2458-14-881 (PMC4159554; doi:10.1186/1471-2458-14-881)
Supplement: Supplementary file 3 — Additional file 3: Table S2: Studies of alcohol and policy interventions to reduce IPV that did not meet design criteria but that provided evidence of mediation of alcohol consumption on IPV. (DOCX 26 KB) [file 12889_2014_7007_MOESM3_ESM.docx]

**Additional file 3: Table S2. Studies of alcohol and policy interventions to reduce IPV that did not meet design criteria but that provided evidence of mediation of alcohol consumption on IPV**

| **Author (date), country, study design** | **Study aim** | **Description of intervention and outcome measures** | **Population/sample** | **Reported results for IPV, alcohol use, other relevant outcomes and mediation** |
| --- | --- | --- | --- | --- |
| **Community-level interventions: Alcohol outlet density** | | | | |
| Liang & Chikritzhs (2011)^48^; Australia; cross-sectional design | To investigate the effect of outlet numbers and alcohol sales on the risk of assault in Western Australia for period 2000/2001. | Alcohol outlet density – numbers of outlets and wholesale volume of alcohol sold by outlet. Geographical unit: local government level.  ***IPV measure:*** Violent assault offences reported to police categorized by location (assaults at private residences proxy for IPV). | Western Australia, population approximately 1.9 million in 2000/2001. | Increase in alcohol sales volume from off-premises outlets associated with increased risk of violence in private residences - incident rate ratio IRR 1.261 (95% CI: 1.11, 1.43*,* *p*<0.05). For every 10,000 additional litres of pure alcohol sold by an off-site outlet, the risk of violence in residential premises increased by 26%. No association with *numbers* of outlet (density) and assaults on residential premises.  No association between IPV and on-premises outlet density or alcohol sales volume.  ***Mediation:*** Interpreting alcohol sales as a proxy for consumption suggests support for mediating effect of alcohol consumption in the relationship between outlet density and IPV. |
| McKinney et al (2009)^49^; USA; multi-level cross-sectional design | To investigate whether alcohol outlet density is associated with male to female partner violence (MFPV) and female-to-male partner violence (FMPV), and whether this association is stronger for risky drinkers (binge drinkers or with alcohol problems) | Alcohol outlet density of the zip code where survey participants resided measured as number of outlets per 10 000 persons (1997 licensing records) divided by total population size (1990 U.S. Census).  ***IPV measure:*** Couple-level measures from 1995 national population survey of self-reported MFPV and FMPV and physical violence (CTS).  ***Alcohol use***: self-reported binge drinking and alcohol-related problems reported from same survey. | National population-based sample in USA (1,597 married/cohabiting couples) from 1995 survey. | An increase in alcohol outlet density was associated with an increased risk of MFPV violence: OR 1.03 (95% CI: 1.00, 1.05, *p*=0.01). No association found with FMPV.  Increase in *on-premises* outlet density was associated with an increased risk of MFPV OR 1.03 (95% CI: 1.00, 1.05, *p*=0.01). No association between *off-premises* outlet density and either type of partner violence.  ***Mediation:*** The relationship between outlet density and MFPV was stronger for couples who had alcohol problems. |
| Roman & Reid (2012)^50^; USA; cross-sectional design | To test whether the density of alcohol outlets across neighbourhoods is positively associated with police calls for service for domestic violence. | Geographic unit for outlet density: outlets per square mile.  ***IPV measure:*** Number and time of 911 calls to police for domestic violence (1 Jan 2005 – 31 Dec 2006). | District of Colombia, Washington USA.  High crime, metropolitan area. 581,530 residents (2006 census)  Used 431 of 433 block groups; average 573 households and 1,304 residents. High youth (20% under 18yo) and high black population (60%) compared with white (31%). | An increase in off-premises outlet density was associated with an increase in domestic violence (b=0.012 *p*<0.001) but an increase in on-premises outlet density was associated with decrease in 911 calls to police for domestic violence (*b*= -0.005 *p*<.001).  ***Mediation:*** stronger relationship between off-premises outlet density and IPV on weekends (*b*=.003 *p*<0.01) suggesting that effect of density is greater during times when heavier drinking more likely to occur. |
| **Relationship-level interventions: Couples-based treatment** | | | | |
| O’Farrell and Murphy (1995)^57^; O’Farrell et al (1999)^58^; O’Farrell et al (2000)^59^; USA; pre-post design; non-concurrent comparison group. | To examine the prevalence and frequency of marital violence in male alcoholics and their wives after Behavioral Marital Therapy (BMT) 12-months and 24-months post treatment. To examine the frequency and prevalence of verbal aggression in male alcoholics and their wives 24-months after BMT. | ***Intervention:*** Behavioral Marital Therapy (outpatient, couples-based).  ***IPV measures:*** Self-reported violence (CTS); verbal aggression (CTS).  ***Alcohol use:*** Self report (TLFB). | 88 male alcoholics and wives treated at Veterans Affairs Medical Clinic, and two year follow up of 75 of 88 participants.  Mean age: males 43.5 (SD 9.0) females 41.6 (9.7)  Ethnicity: White 98.9% males, 98.9% females.  Length of relationship: 13.9 years (SD 9.9).  Compared IPV with demographically matched non-alcoholic comparison sample from 1985 National Family Violence Re-Survey. | Prevalence and frequency of violence significantly decreased in 12- and 24 months after BMT compared to 12 months before BMT but remained significantly elevated relative to matched non-alcoholic sample. No change between first and second year.  Significant decreases for both alcoholic men and their wives in verbal aggression in first and second year after BMT though violence levels remained elevated relative to matched normal comparison sample.  ***Mediation:*** Remitted alcoholics no longer had elevated domestic violence levels whereas relapsed alcoholics did. Frequency of violence correlated with number of days drinking.  Relapsed alcoholics and wives more verbally aggressive than remitted alcoholics and comparison sample. |
| O’Farrell et al (2004)^60^; USA; pre- and post-test design; non-concurrent comparison group. | To examine partner aggression among male alcoholic patients and their female partners in the year before and two years after Behavioral Couples Therapy. | ***Intervention:*** Behavioral Couples Therapy (BCT).  12- and 24- month follow up.  ***IPV measure:*** Self-reported verbal aggression and violence (CTS).  ***Alcohol use:*** Self-report (TLFB). | 303 male alcoholic patients and female partners. Recruited from four project sites where couples had signed up to participate in Counseling for Alcoholics’ Marriages (CALM) program.  Mean age: males 43.3 (SD 10.0) females 41.1 (9.9)  Ethnicity: White 95.4% males, 96.4% females.  Length of relationship: 13.2 years (SD 10.7).  Compared IPV with demographically matched non-alcoholic comparison sample from 1985 National Family Violence Re-Survey. | Partner aggression and violence decreased in first and second year after BCT from year before BCT but still higher than comparison sample.  ***Mediation:*** Clinically significant violence reductions in patients whose alcoholism was remitted after BCT (violence reduced to almost same level as comparison sample and 30% less than relapsed patients). |
| Rotunda et al (2008)^61^; USA; pre- and post-test design; no comparison group. | To compare drinking, relationship and psychological distress outcomes before and after BCT for male veterans. | ***Intervention:*** Behavioral Couples Therapy (BCT) (outpatient).  12-month follow up.  ***IPV measure:*** Self-reported male-to-female violence (CTS). | 38 male alcohol dependent veterans with PTSD (*n*=19) or without PTSD (*n*=19) and female partners recruited from Veterans Affairs Outpatient BCT program.  Mean age = PTSD group 48.32 (SD 7.70); without PTSD 48.16 (SD 8.30).  Length of relationship: PTSD group 14.43 (SD 13.03); without PTSD 13.42 (SD 11.47).  Ethnicity: White 94.74% both groups. | ***Mediation:*** After treatment, both groups showed reductions in drinking and negative consequences of drinking, increased relationship satisfaction and decrease in frequency of male-to-female violence. |
| Schumm et al (2009)^62^; USA; pre- and post-test design; non-concurrent comparison group. | To examine partner violence before and 12- and 24-months after BCT. | ***Intervention:*** Behavioral Couples Therapy (BCT).  12- and 24-month follow up.  ***IPV measures:*** Self-reported male and female-perpetrated verbal aggression, overall violence, and severe violence (CTS).  **Alcohol use:** Self-report (TLFB). | 103 female alcoholic patients and male partners recruited from four project sites where couples had signed up to participate in Counseling for Alcoholics’ Marriages (CALM) program.  Mean age = 39.96 years (SD 8.10)  Relationship length 11.17 (SD 9.46).  Ethnicity: White (92%).  Compared IPV with demographically matched non-alcoholic comparison sample from 1985 National Family Violence Re-Survey. | Before BCT, female alcoholic patients and male partners had elevated violence levels compared to non-alcoholic comparison group. In first and second year after BCT, female-perpetrated violence decreased significantly from before BCT. Male partner aggression also significantly reduced in first and second year after BCT, except for prevalence and frequency of severe violence at 12-months.  ***Mediation:*** Women and men’s aggression generally significantly lower for remitted than relapsed cases (to level of matched comparison) though reductions did not reach significance in second year; no difference between groups for severe violence by both partners. |
| **Individual-level interventions: Treatment** | | | | |
| Mignone et al (2009)^71^; USA; post-treatment survival analysis. | To investigate whether time to relapse to violence was related to male partner’s relapse to alcohol after treatment.  Also considered the moderating effects of female partner drinking and anti-social personality disorder (ASPD). | ***Intervention:*** Individual-based alcoholism treatment (outpatient).  12-month follow up.  ***IPV measures:*** Self-reported physical aggression (CTS; TLFB-SV); survival analysis to assess time to relapse to violence.  ***Alcohol use:*** Daily drinking log completed by both partners. | 147 male alcoholic IPV perpetrators and non-alcoholic female partners recruited from alcoholism treatment program.  Mean age males = 32.1 years (SD 8.9); females 30.7 years (SD 7.7).  Ethnicity (males): Caucasian 54%; African-American 27%; Hispanic 13%. | ***Mediation:*** Those who relapsed to alcohol were much more likely to relapse to physical aggression. Odds of any male-to-female partner violence was more than 3.7 times and odds of severe violence 6 times greater for those who relapsed than those who did not.  Female alcohol consumption increased the likelihood of victimization depending on her level of consumption; heavier consumption increased the risk of experiencing severe violence  Significantly stronger relationship between alcohol use and non-severe violence among men diagnosed with ASPD; not significant for severe violence. |
| O’Farrell et al (2003)^72^; USA; pre-post design; non-concurrent comparison group. | To examine partner violence in the year before and year after alcoholism treatment for male alcoholic patients. | ***Intervention:*** Individual-based alcoholism treatment (outpatient).  Comparison 12-months prior and 12-months post treatment.  ***IPV measures:*** Self-reported male and female-perpetrated violence and verbal aggression (CTS).  ***Alcohol use:*** Self report (TLFB). | 301 male alcoholics entered into treatment in two outpatient clinics.  Mean age: males 42.1 (SD 12.6) females 39.2 (12.6)  Ethnicity: White 80.7% males, 79.1% females.  Length of relationship: 10.2 years (SD 8.0).  Compared IPV with demographically matched non-alcoholic comparison sample from 1985 National Family Violence Re-Survey. | Violence towards wives decreased significantly from 56% to 25% but still higher than comparison group.  Significant increase in percentage days abstinent.  ***Mediation:*** Couples where alcoholic patient relapsed had significantly greater verbal aggression and overall violence than remitted patients. No difference between groups for severe violence or males’ elevated verbal aggression. |
| Taft et al (2010)^73^; USA; pre- and post test; no comparison group | To examine static and time-varying risk factors for perpetration of IPV among men in alcohol treatment. | ***Intervention:*** Standard individual-based alcoholism treatment (inpatient, outpatient).  6- and 12-month follow up.  ***IPV measure:*** Self-reported male physical aggression (CTS2).  ***Alcohol use:*** Self-report (TLFB). | 178 male alcoholics (and female partners) with IPV perpetration at baseline (*n*=75) and without (*n*=103). Recruited from alcoholism treatment program.  Mean age = 41.0 years (SD 8.5).  Years living together = 10.7 (SD 9.1)  Ethnicity: European American (85%). | Those who reported IPV at baseline showed significant declines in IPV following treatment (43% at 6-months and 36% at 12-months). For those without baseline IPV, new incidence of IPV 15% at 6-months and 7% at 12-months.  ***Mediation:*** Alcohol use was not associated with IPV recurrence among those who reported IPV at baseline. However alcohol use was associated with new incidents of IPV among those without prior reported IPV. |

AUDIT – Alcohol Use Disorders Identification Test

CTS – Conflict Tactics Scale

CTS2 – Revised Conflict Tactics Scale

TLFB – Timeline Follow Back Interview

TLFB-SV – Timeline Follow Back Interview for Spousal Violence
